# Genome-Wide Analysis of the *PERK* Gene Family in *Brassica napus* L. and Their Potential Roles in Clubroot Disease

**DOI:** 10.3390/ijms26062685

**Published:** 2025-03-17

**Authors:** Zeyu Zhang, Tongyu Fu, Cong Zhou, Fan Liu, Lingyi Zeng, Li Ren, Chaobo Tong, Lijiang Liu, Li Xu

**Affiliations:** The Key Laboratory of Biology and Genetic Improvement of Oil Crops, The Ministry of Agriculture and Rural Affairs of the PRC, Oil Crops Research Institute, Chinese Academy of Agricultural Sciences, Wuhan 430062, China; 20241015050@stu.scau.edu.cn (Z.Z.); futongyv@163.com (T.F.);

**Keywords:** *Brassica napus*, *PERK* gene family, receptor-like kinase, hormones, clubroot disease

## Abstract

The proline-rich extensin-like receptor kinase (*PERK*) gene family is crucial to various molecular and cellular processes in plants. We identified 50 *PERK* genes in *Brassica napus* to explore their evolutionary dynamics, structural diversity, and functional roles. These genes were grouped into four classes and unevenly distributed across 18 chromosomes. Phylogenetic studies and Ka/Ks ratios revealed purifying selection during the evolution process. They exhibited significant diversification in gene length, molecular weight, and isoelectric points, suggesting specialized function. Gene structure and motif analyses revealed variations among the *BnPERK* family members, with conserved tyrosine kinase domains suggesting functional importance. *Cis*-element analysis predicted the involvement in hormone signaling and stress responses. Expression profiling showed diverse patterns across tissues and hormone treatments, highlighting potential roles in growth regulation and hormone signaling. Protein–protein interaction networks suggested BnPERK proteins interact with a wide array of proteins, implicating them in multiple biological processes. The transcriptional downregulation of four *BnPERK* genes upon *Plasmodiophora brassicae* infection implied a role in clubroot disease response. Furthermore, the Arabidopsis *perk9* mutant displayed relieved disease severity and enhanced basal immune response, suggesting the negative role of *PERK9* in plant immunity. The study highlighted the potential role of *BnPERKs* in crop improvement strategies against clubroot disease.

## 1. Introduction

Receptor-like kinases (RLKs) constitute the largest plant receptor family and are located on the cell surface to perceive and transduce a wide range of signals [1]. By initiating intracellular signaling cascades, RLKs play pivotal roles in various molecular and cellular processes, including plant development, stress response, and signal transduction [2]. These versatile receptors are capable of sensing plant hormones, secreted peptides, and molecules derived from pathogens involved in plant defense response [3]. For example, RLKs recognize pathogen-associated molecular patterns (PAMPs), which trigger the Pattern-Triggered Immunity (PTI) response, activating signaling pathways that defend against pathogen invasion.

RLKs typically consist of three key domains: an extracellular domain, a transmembrane domain, and an intracellular kinase domain [4]. The extracellular domain, characterized by significant structural diversity, forms the basis for classifying RLKs into different subclasses based on their motif structures. Among these, the proline-rich extensin-like receptor kinases (PERKs) represent a distinct group characterized by a proline-rich extracellular domain [5]. Interestingly, while some *PERKs* like *BnPERK1* and *AtPERK4* locate on the plasma membrane, *AtPERK13* has been demonstrated to anchor to the cell wall [6,7,8,9]. Functionally, the intracellular kinase domain of RLKs contains conserved motifs that exhibit kinase activity, which has been observed in *BnPERK1* and *AtPERK4* [7,10].

In *Arabidopsis thaliana*, the *PERK* gene family consists of 15 members, some of which show specific expression patterns in roots and floral organs [11]. However, some *AtPERK* genes exhibit broad expression patterns across various tissues, such as *AtPERK1,* which is ubiquitously expressed, suggesting its involvement in diverse molecular and cellular processes. Recent studies have elucidated the regulation of *PERKs* in response to different stress conditions [5]. For example, the expression of *TaPERKs* in wheat and *GhPERKs* in cotton is significantly influenced by abiotic stresses such as heat, drought, and salinity [12,13]. In *A. thaliana*, *AtPERK13* has been shown to respond to nutrient deficiencies such as phosphate, nitrogen, and iron deprivation, highlighting the role of *PERKs* in maintaining nutrient homeostasis and adaptation [9]. In rice, the *PERK* gene Z15 is activated by moderately low temperatures, emphasizing the adaptability of *PERKs* to diverse environmental cues [14]. Additionally, *PERKs* have been implicated in plant defense against pathogens. For example, the expression of *TaPERKs* is influenced by fungal pathogens, and *BnPERK1* has been shown to respond to the pathogen *Sclerotinia sclerotiorum* [7,12,15]. In summary, *PERKs* are versatile players in response to various stresses.

*PERKs* also play essential roles in plant hormone signaling. Several studies have highlighted the involvement of *PERKs* in the regulation of growth and development in response to hormonal signals [10,13,16]. For instance, *AtPERK4* has been implicated in abscisic acid (ABA) signaling during root cell elongation [10]. Furthermore, the expression profiles of *OsPERKs* and *GhPERKs* have shown differential expression patterns under different plant hormone treatments, including ABA, methyl jasmona (MeJA), gibberellin (GA), salicylic acid (SA), and auxin [13,16]. The intricate regulation of *PERKs* by different plant hormones suggests their pivotal role in integrating hormonal signaling pathways to coordinate plant growth and stress responses.

In summary, *PERK* genes are known to be critical to plant growth, development, and stress responses. Despite the essential roles of *PERKs* in various processes across various plant species, comprehensive studies on the *PERK* gene family in *Brassica napus* (oilseed rape), an economically important crop serving as both an edible oil source and animal feed, are still limited [17]. This allopolyploid species (AACC, 2n = 38), formed by the hybridization between two diploid ancestors, *B. rapa* (AA = 20) and *B. oleracea* (CC = 18), emerged approximately 6800 to 12,500 years ago [18]. While a comprehensive study of *BrPERK* genes has been completed in male-sterile *B. rapa* plants, identifying 25 *BrPERK* genes and exploring their potential role in male sterility, studies in *B. napus* remain scarce [19].

This study aimed to provide a comprehensive study of *BnPERK* genes to include the exploration of its evolutionary history, chromosome location, and gene structure through various computational approaches. Tissue-specific and hormone treatment gene expression patterns were examined using available transcriptome datasets. Additionally, the study also investigated the role of *BnPERKs* in response to the obligate pathogen *Plasmodiophora brassicae,* which causes clubroot disease in *Brassica* species. By providing insights into the molecular and functional roles of *BnPERKs*, this study will contribute to a deeper understanding of this often-overlooked plant receptor subfamily and its potential applications in crop improvement.

## 2. Results

### 2.1. Identification and Phylogenetic Analysis of BnPERKs in Brassica napus

Fifty *PERK* genes were identified within the *B. napus* ‘ZS11’ genome (Supplementary Tables S2 and S3). Identification was accomplished through an extensive BLAST search using 15 *AtPERK* sequences, revealing a higher number of *BnPERK* genes compared with those reported in *Arabidopsis*, Chinese cabbage, soybean, rice, sorghum, maize, cotton, and wheat (Supplementary Table S4). The BnPERK family of proteins exhibited varying lengths, ranging from 187 to 921 amino acids. Their molecular weights (MWs) spanned from 20.63 to 102.21 kDa, with BnPERK15C2 and BnPERK15A2 representing the extremes. Additionally, the isoelectric points (pIs) ranged from 5.26 to 8.59 (Supplementary Table S5). The data plots illustrated that BnPERK proteins exhibit a broad spectrum of MW and pI. Moreover, predictions of subcellular localization suggested that most BnPERK proteins were located on the plasma membrane and endomembrane system, while others were associated with the nucleus and chloroplast (Supplementary Table S5).

**Figure 1:**
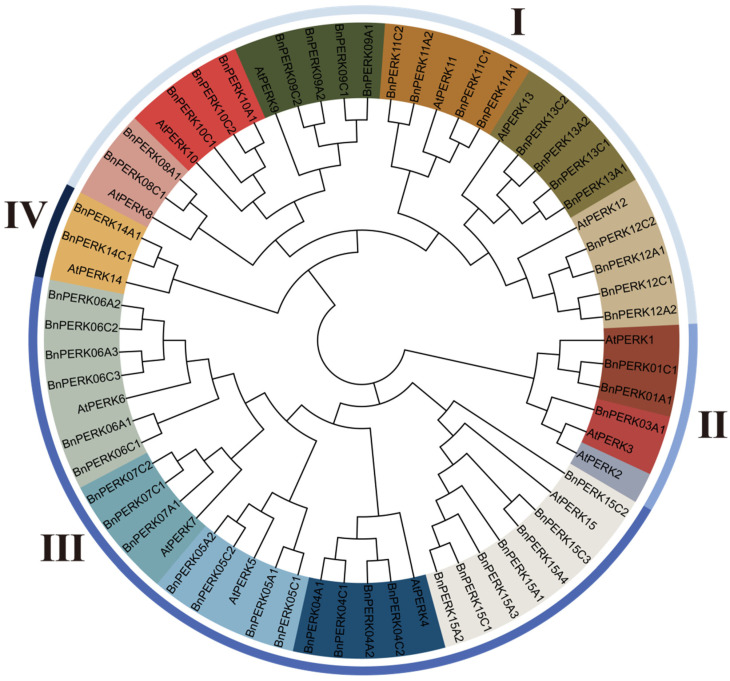
Phylogenetic tree of the *PERK* gene family in *B. napus* and *A. thaliana*. The phylogenetic tree was constructed with 50 BnPERK protein sequences and 15 AtPERK protein sequences by MEGA11 using the maximum likelihood (ML) method. A total of 15 paralogs were identified, grouped into 15 distinct clusters, each represented by a different color (PERK1 to PERK15). Based on sequence similarity and functional domain analysis, the 15 paralogs were further categorized into four major groups, which are indicated by distinct colors (outer lane). Group I, light steel blue; Group II, cornflower blue; Group III, royal blue; Group IV, black.

To investigate the phylogenetic distribution and evolutionary relationships of the *PERK* gene family, a phylogenetic tree was constructed using 50 BnPERK protein sequences and 15 AtPERK protein sequences by MEGA11 using the maximum likelihood (ML) method (Supplementary Table S6). The *BnPERK* genes were categorized into four groups (I–IV) based on sequence similarity and functional domain analysis. Group III, being the largest group, included 5 paralogs with 24 *BnPERK* genes. Group I followed, containing 6 paralogs with 21 *BnPERK* genes. Group II included 3 paralogs, with 3 *BnPERK* genes, while Group IV contained only 1 paralog, with 3 *BnPERK* genes (Figure 1). All *AtPERK* genes, with the exception of *AtPERK2*, exhibited a close syntenic association with the 50 *BnPERK* genes. Most *AtPERK* genes corresponded to 2–6 orthologous genes in *B. napus*, each comprising orthologous genes from both the A and C subgenomes of *B. napus*. Notably, *AtPERK3* was associated with a single ortholog, *BnPERK03A1*, as its counterpart in the C subgenome appeared to have been lost. These results suggest that genome rearrangement and gene loss occurred during the polyploidization of *B. napus*.

### 2.2. Chromosomal Distribution and Collinearity Analysis of BnPERK Genes

**Figure 2:**
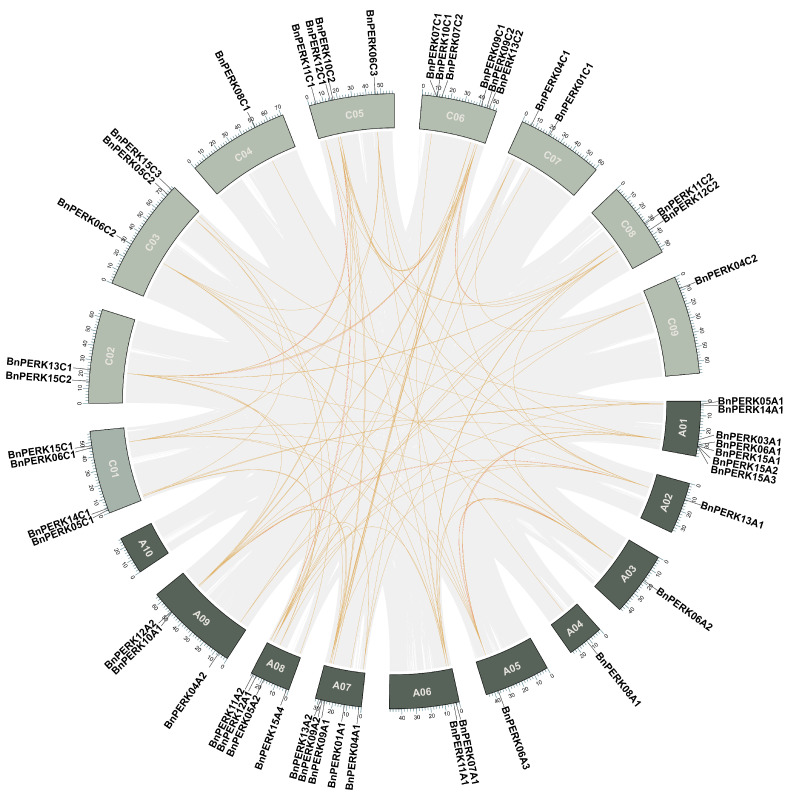
Chromosomal distribution and collinearity relationships of *BnPERK* genes. The locations of all the chromosomal *BnPERK* genes are shown in different chromosomes. The light green boxes stand for A subgenomes and dark green boxes stand for C subgenomes in *Brassica napus*. The light brown lines represent collinearity in *BnPERK* gene pairs.

The 50 *BnPERK* genes were evenly distributed across the A and C subgenomes of *B. napus*, with 25 genes in each (Figure 2 and Supplementary Figure S1). These genes were dispersed among all chromosomes, except for A10. The highest concentration of genes was on the chromosome A1, which harbored seven genes, followed by six genes on C6 and five genes on A7. Single *BnPERK* genes were identified on chromosomes A2, A3, A4, A5, C4, and C9. The results indicated an irregular arrangement of the *PERK* gene family across the rapeseed chromosomes (Figure 2).

The collinear gene pairs in the *B. napus* genome were analyzed to explore syntenic relationships among *BnPERKs* (Figure 2). We identified 81 collinear gene pairs, including 49 pairs resulting from segmental duplication between the A and C subgenomes and 32 pairs occurring within a subgenome. The result indicated a high conservation between the A and C subgenomes. Additionally, we assessed the selective pressure exerted on duplicated *BnPERK* genes by calculating the ratios of nonsynonymous to synonymous substitutions (Ka/Ks) for the paralogous gene pairs (Supplementary Table S7). The Ka/Ks ratios ranged from 0.1409 to 0.2518 for 17 gene pairs in the A subgenome and from 0.1682 to 0.2519 for 15 gene pairs in the C subgenome. Furthermore, the Ka/Ks ratios for 49 gene pairs between the A and C subgenomes varied from 0.0482 to 0.5148. Notably, the Ka/Ks ratios for all paralogous gene pairs were found to be less than 1, indicating purifying selection acting on *BnPERK* genes.

### 2.3. Gene Structure, Conserved Motifs, and Cis-Elements Analysis of BnPERK Genes

**Figure 3:**
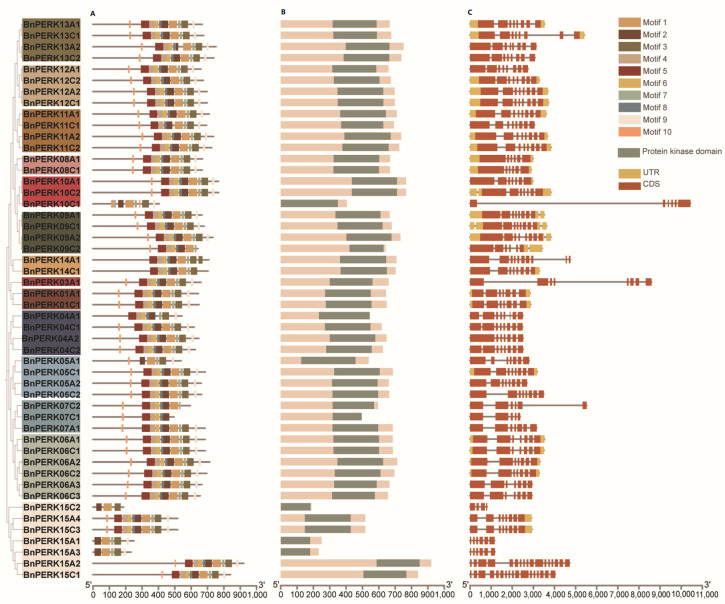
Phylogenetic tree, conserved motifs, and gene structure analysis of *BnPERK* gene family. A total of 14 paralogs were identified based on the phylogenetic tree, each represented by a different color. (**A**) Motif composition of BnPERK proteins, with motifs 1–10 displayed in different colored boxes. (**B**) Identification of the protein kinase domain in BnPERK proteins. (**C**) Exon–intron analysis of *BnPERK* genes, with yellow boxes representing un-translated regions (UTRs) and orange boxes represented coding sequences (CDSs).

To elucidate the functional characteristics of *BnPERKs*, the conserved motif, protein domain, and exon–intron organization were analyzed together with the phylogenetic tree (Figure 3). Ten conserved motifs were identified across the BnPERK proteins (Figure 3A and Supplementary Figure S2). These motifs exhibited consistent distribution patterns within the BnPERK proteins, with the exceptions of BnPERK15C2, BnPERK15A1, BnPERK15A3, and BnPERK10C1. Moreover, all *BnPERK* genes shared conserved tyrosine kinase domains, highlighting their functional significance (Figure 3B). Exon–intron analysis revealed structural diversity within the *BnPERK* gene family, though orthologous genes generally shared similar gene structures (Figure 3C). Most *BnPERK* genes harbored 5–9 introns, with the number of introns ranging from 3 (*BnPERK15C2*) to 16 (*BnPERK15A2*).

**Figure 4:**
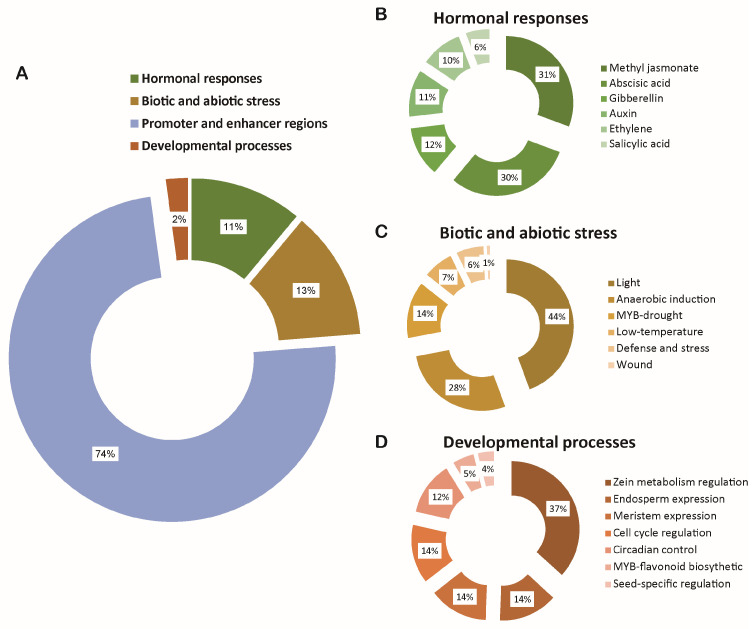
*Cis*-elements analysis of the *BnPERK* promoters. The *cis*-elements were predicted by analysis of 2 Kb upstream regions of *BnPERK* genes. They were categorized into four groups (**A**), including hormonal responses (**B**), biotic and abiotic stress (**C**), developmental processes (**D**), and promoter and enhancer regions, which are represented by different colors and further categorized.

*Cis*-elements analysis is crucial for understanding gene regulation and function. Accordingly, upstream 2 kb sequences from *BnPERKs* transcription start sites were analyzed (Supplementary Tables S2 and S8). The results revealed that 75% of the identified *cis*-elements were core promoter and enhancer region elements. The remaining *cis*-elements were associated with hormonal responses, various stresses, and developmental processes (Figure 4A and Supplementary Figure S3). *Cis*-elements regulated responses to diverse hormones, including methyl jasmona (MeJA), abscisic acid (ABA), gibberellin (GA), auxin, ethylene (ET), and salicylic acid (SA), which were calculated (Figure 4B). The ABA and MeJA responsive elements were abundantly detected, and the auxin responsive elements were secondarily enriched (Figure 4B). These *cis*-elements relate to various biotic and abiotic stresses, such as light, anaerobic conditions, drought, low temperature, defense, and wounds (Figure 4C). Notably, there were light-responsive elements in all of the *BnPERK* genes (Supplementary Figure S3). In addition, there was a small part of the *cis*-elements that were categorized according to their roles in diverse developmental processes, including zein metabolism, endosperm expression, meristem expression, cell cycle regulation, and circadian control (Figure 4D). The presence of such diverse *cis*-elements suggests potential regulatory roles of *BnPERKs* in diverse biological processes.

### 2.4. Expression Pattern of BnPERK Gene Family Across Various Tissues

**Figure 5:**
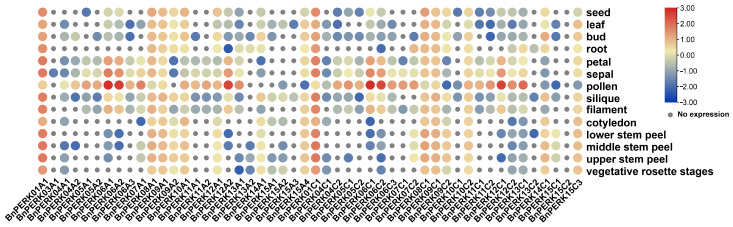
Expression profiles of the *BnPERK* gene family in various tissues of *Brassica napus*. The heatmap shows the expression of 50 *BnPERK* genes across 14 different tissues, processed with log_2_ normalization. The color gradient ranges from blue (indicating lower expression levels) to red (indicating higher expression levels), while the gray spots represent no detectable expression.

The expression pattern of the *BnPERK* genes was analyzed across 14 tissues at various developmental stages through RNA-Seq analysis (Figure 5). The results exhibited diverse expression patterns across these tissues. There were 13 genes showing constitutive expression in multiple tissues, among which *BnPERK01A1* and *BnPERK01C1* exhibited the highest expression levels. In contrast, certain genes displayed specific expression patterns. For example, *BnPERK03A1* showed highly specific but relatively low expression in the sepal, and *BnPERK15A3* specifically expressed in the leaf and silique. Moreover, 18 orthologous genes of *AtPERK5*, *AtPERK6*, *AtPERK7*, and *AtPERK12* in *B. napus* were highly expressed in pollen. *BnPERK15C2*, which encoded the shortest protein, was not expressed in any tissues, suggesting a potential loss of function. These differential expression patterns of *BnPERK* genes point to their diverse roles in plant growth and development.

Furthermore, some paralogous gene pairs, such as *BnPERK01A1*/*BnPERK01C1* and *BnPERK09A1*/*BnPERK09C1*, showed similar expression patterns, being constitutively and highly expressed across all tested tissues. In contrast, other paralogous gene pairs, like *BnPERK06A1*/*BnPERK06A2* and *BnPERK06C1*/*BnPERK06C2*, showed specific high expression in floral organs. Additionally, *BnPERK15A4*/*BnPERK15C3* showed high expression levels in different tissues, except for pollen. However, some paralogous gene pairs exhibited distinct patterns, indicating functional divergence.

### 2.5. Expression Pattern of BnPERK Gene Family in Response to Hormone Treatments

**Figure 6:**
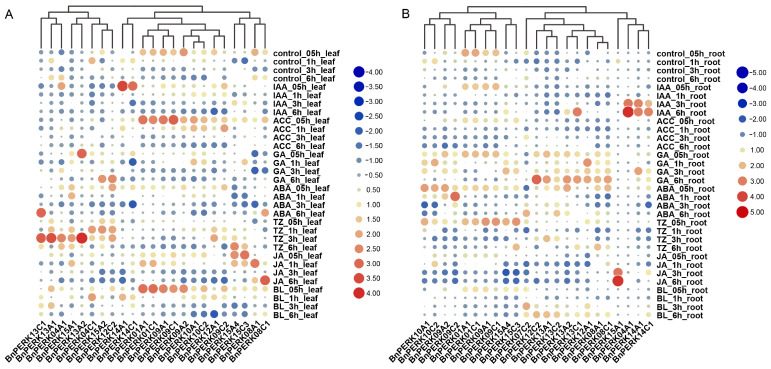
Expression patterns of *BnPERKs* under hormone treatments in leaves (**A**) and roots (**B**). The heatmap displays the relative expression of *BnPERK*s at 0.5, 1, 3, and 6 h after treatment, derived from RNA-Seq analysis. IAA, indole acetic acid; GA, gibberellin acid; ABA, abscisic acid; ACC, l-aminocyclopropane-l-carboxylic acid; TZ, trans-zeatin; JA, methyl jasmonate; BL, brassinolide. Different dot colors represent different relative expression levels: red dot indicates up-regulated expression, blue dot indicates down-regulated expression, and the larger the dot, the greater the fold change.

In order to further explore the function of *BnPERK*s during hormone responses, we investigated their expression patterns in both leaves and roots after different hormone treatments (Figure 6). Among the 50 identified *BnPERK* genes, only a small subset was expressed in leaves and roots. Most of these expressed genes were strongly induced in leaves within 0.5 h after indole acetic acid (IAA), l-aminocyclopropane-l-carboxylic acid (ACC), gibberellin acid (GA), trans-zeatin (TZ), or brassinolide (BL) treatments (Figure 6A). Additionally, four genes—*BnPERK15A4*, *BnPERK15C3*, *BnPERK08A1*, and *BnPERK08C1*—showed enhanced transcript levels at different time points after methyl jasmonate (MeJA) treatment in leaves. In contrast, their expression patterns in roots were distinct. Most genes showed enhanced expression levels after GA and ABA treatments but decreased expression levels after MeJA treatment (Figure 6B). Notably, *BnPERK15A1* was strongly induced after MeJA treatment. Furthermore, three genes, *BnPERK09A1*, *BnPERK09C1*, and *BnPERK04C1*, which showed increased expression in leaves after IAA treatment, were also induced in roots.

### 2.6. Protein–Protein Interaction Network Analysis of BnPERK Genes

**Figure 7:**
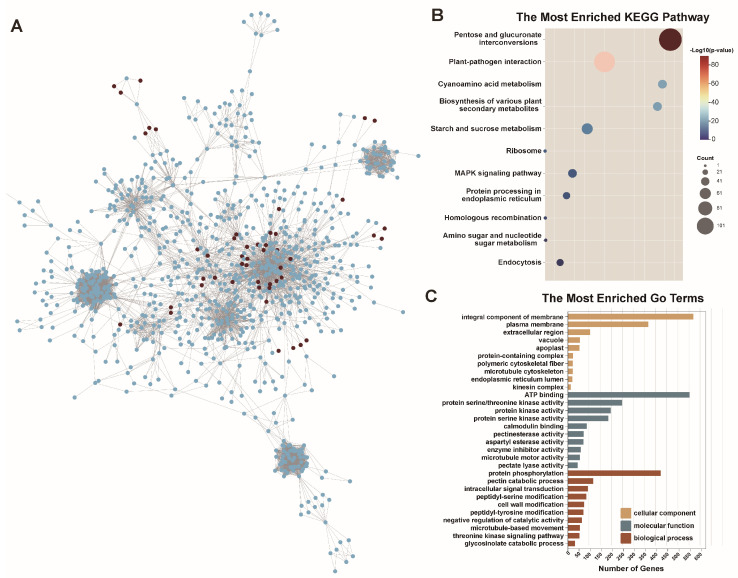
Proteins interacting with BnPERK proteins in *Brassica napus*. (**A**) Protein–protein interaction network of BnPERK proteins. Dark brown spots represent BnPERK proteins, blue spots represent proteins that interact with BnPERK proteins, and gray lines indicate the interaction between BnPERK proteins and other proteins. (**B**) KEGG enrichment analysis of proteins that interacted with BnPERK proteins. (**C**) GO enrichment analysis of proteins that interacted with BnPERK proteins.

Predictive interaction networks were employed to explore the biological mechanisms of BnPERK proteins. The analysis revealed that the BnPERK proteins putatively interact with 1133 proteins in *B. napus*. The results showed that most PERK proteins serve as core nodes in these networks, interacting with various proteins participating in different biological processes (Figure 7A and Supplementary Table S9).

KEGG enrichment analysis indicated that the interacting proteins were primarily involved in processes such as pentose and glucuronate interconversions, plant–pathogen interaction, starch and sucrose metabolism, cyanoamino acid metabolism, the biosynthesis of secondary metabolites, endoplasmic reticulum processing, and the MAPK signaling pathway (Figure 7B and Supplementary Table S10). Similarly, GO enrichment analysis revealed significant enrichment of terms like integral components of the membrane and plasma membrane in the cellular component category (Figure 7C and Supplementary Table S11). ATP binding and kinase activity were prominently enriched in the molecular function category. Biological process category terms such as protein phosphorylation, pectin catalytic activity, and cell wall modification were enriched. Protein interactions analysis provided a comprehensive view of the BnPERK proteins interaction network in *B. napus*, underscoring their central role and broad involvement in different biological processes, including sugar metabolism and plant–pathogen interaction.

### 2.7. Root Gall Formation and BnPERK Gene Expression in B. napus upon Plasmodiophora brassicae Inoculation

**Figure 8:**
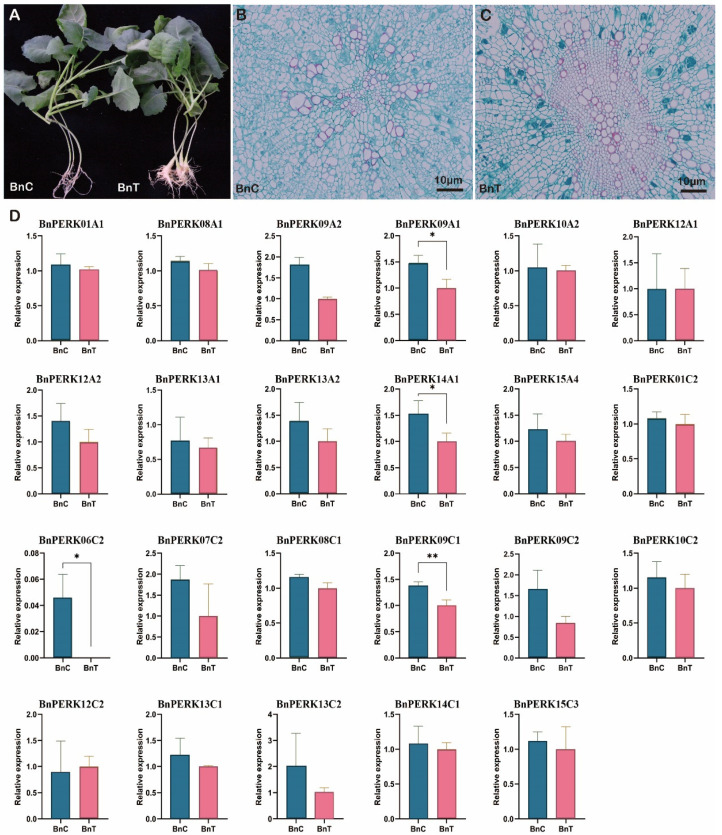
Disease symptoms, histocytological analysis, and *PERK* gene expression analysis in control and inoculated *B. napus*. (**A**) Symptoms of clubroot disease in control and inoculated roots at 28 days after inoculation. (**B**,**C**) Histocytological analysis of cross-sections of control and inoculated roots at 28 days after inoculation. (**D**) Expression patterns of 23 *PERK* genes at 14 days after inoculation with *Plasmodiophora brassicae*. Error bars represent the standard error of the means of the three replicates. Stars indicate significant differences in expression between control and inoculated roots (|log_2_ (fold-change)| > 1). *, *p* < 0.05; **, *p* < 0.01.

Galls were observed on the roots of *B. napus* at 28 days after inoculation with *P. brassicae* (Figure 8A). Cross-sections of the inoculated roots revealed the changes in the vascular system. Resting spores that stained blue formed in the diseased roots. Furthermore, diseased root cells exhibited hypertrophy and disorder, accompanied by thickened cell walls, while control roots grew normally (Figure 8B). To better understand the response of *BnPERK* genes to clubroot disease, we analyzed the expression of *BnPERK* at 14 days after *P. brassicae* inoculation by RNA-Seq (Figure 8C). Among the 50 identified *BnPERK* genes, 23 were expressed in either inoculated or control roots. Notably, four genes, namely *BnPERK09A1*, *BnPERK09C1*, *BnPERK14A1*, and *BnPERK06C2,* showed downregulated expression in response to *P. brassicae*, among which *BnPERK06C2* was completely inhibited after inoculation. Additionally, the paralogous gene pair *BnPERK09A1*/*BnPERK09C1* also exhibited consistent expression patterns across different tissues. These four downregulated *BnPERK* genes were implicated in the response to *P. brassicae*.

### 2.8. Function of AtPERK9 in Clubroot Disease

**Figure 9:**
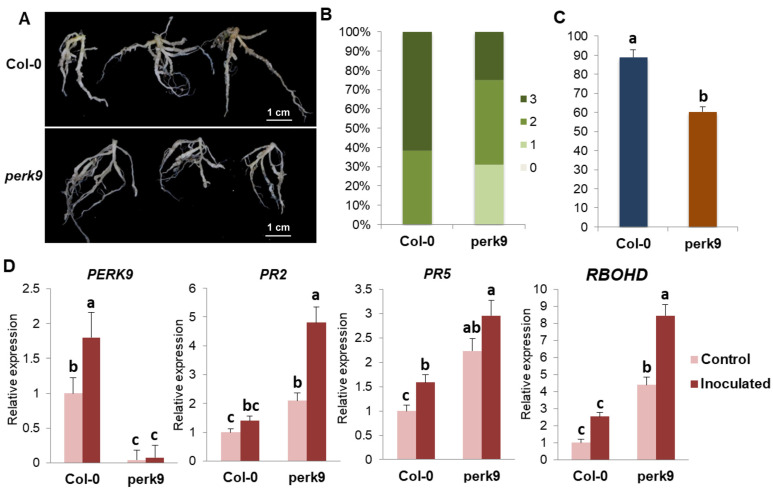
Enhanced disease severity of clubroot disease in the mutant of *AtPERK9*. (**A**) Root phenotypes of wild-type *A. thaliana* (Col-0) and *perk9* at 28 days after inoculation. (**B**) The occurrence of clubroot disease in Col-0 and *perk9* was investigated according to the 0–3 scoring system. (**C**) Disease index of Col-0 and *perk9*. (**D**) Expression patterns of *PERK9*, *PR2, PR5,* and *RBOHD* genes in Col-0 and *perk9* lines. Different letters represent significant differences (*p* < 0.05).

To explore the putative role of *BnPERK09A1* and *BnPERK09C1* in clubroot disease, the mutant of the orthologous gene *AtPERK9* (At1g68690) was used for clubroot disease investigation. Both the Col-0 and *perk9* showed disease symptoms on the main roots and the lateral roots (Figure 9A). The percentage of severely diseased plants (disease grade 3) in Col-0 was much higher than that in *perk9*, which resulted in a significantly higher disease index in Col-0 (Figure 9B,C). It indicated that *perk9* showed decreased severity of the clubroot disease.

Furthermore, the expression levels of *PERK9*, *PR2*, *PR5,* and *RBOHD* were evaluated in Col-0 and *perk9* lines at 14 days after *P. brassicae* inoculation. *PERK9* also showed downregulated expression in response to *P. brassicae* in Col-0, while it was totally inhibited in *perk9*. The expressions of *PR2*, *PR5*, and *RBOHD* genes were greatly enhanced in *perk9*. *PR2* and *RBOHD* not only could be induced in Col-0 after *P. brassicae* inoculation, but also in *perk9*. Enhanced disease resistance to clubroot disease and increased expression of these genes in *perk9* indicated that the basal immune response was triggered by the knockout of *PERK9.*

## 3. Discussion

PERKs constitute a crucial subfamily of RLKs that play important roles in various molecular and cellular processes in plants, including development, signaling, and stress responses [5]. Despite their significance, there remains a lack of comprehensive study on the molecular functions of PERKs, particularly when compared with other RLKs. While genome-wide analyses of *PERK* genes have been conducted in some species [11,12,13,16,19], a thorough study of the *BnPERK* gene family has yet to be published. Therefore, this study systematically analyzed *BnPERK* genes, covering various aspects.

### 3.1. Deciphering the Evolutionary Dynamics of the BnPERK Genes

There were 50 *PERK* genes identified in the rapeseed cultivar ‘ZS11’ genome, highlighting an increased number of *BnPERK* genes compared with previous studies (Supplementary Table S3). This expansion of *BnPERK* genes was attributed to the evolutionary path of the *Brassica* genus, which experienced a genome triplication following its divergence from the *Arabidopsis* lineage [17,18]. Through interspecific hybridization between *B. rapa* (AA) and *B. oleracea* (CC), the allotetraploid *B. napus* (AACC) arose. This complex evolutionary process contributed to the expansion of the *BnPERK* gene family. Studies have consistently demonstrated that whole-genome duplication and segmental duplications are crucial mechanisms driving gene duplication and the expansion of gene families [20,21,22]. The prevalence of paralogous gene pairs resulting from segmental duplication highlights the role of genome duplication events in this expansion.

Initially, it was expected that each *Arabidopsis* gene would have six homologs in *B. napus* due to two recent duplication events. However, only 50 *BnPERK* genes were identified, suggesting a loss of duplicated *BnPERK* genes following *Brassica* genome triplication and hybridization. This indicated that gene loss was a crucial process during genomic arrangements and chromosome doubling [23]. This also implied a selective process, where functionally redundant *BnPERK* copies may have been lost, while the retained 50 *BnPERK* genes may be crucial for plant development, hormone signaling, and stress response.

Phylogenetic analysis revealed that most *AtPERK* genes corresponded to 2–6 orthologous genes in *B. napus*, each of which included orthologs from both the A and C subgenomes. However, some *AtPERK* genes showed variations in the number of corresponding orthologous genes from the A and C subgenomes, suggesting extensive chromosomal rearrangements and asymmetric gene loss within duplicated genomic blocks [17]. Analysis of Ka/Ks ratios of paralogous gene pairs indicated purifying selection on duplicated *BnPERK* genes, implying their functional conservation and evolutionary constraint at these loci [24].

Understanding the evolutionary dynamics of the *BnPERK* gene family not only enriches our knowledge of rapeseed genetics but also has broader implications for research in plant biology and stress-response mechanisms.

### 3.2. Exploring the Structural Diversity and Functional Roles of BnPERKs

The *BnPERK* gene family exhibited significant structural diversity, including variations in gene length, molecular weight, and isoelectric points. These variations suggested potential functional specialization within the gene family [25].

Although functional studies of *BnPERKs* are limited, expression profiling can still provide valuable insights about the gene’s function and its role in different biological processes [26]. In accordance with the result in *A. thaliana*, *BnPERK* family members exhibited two distinct expression patterns: some were highly expressed in pollen, while others had broader expression patterns [11]. Constitutive expression of certain *BnPERK* genes indicated their essential roles in fundamental cellular functions, while some *BnPERKs* exhibited higher expression levels in floral organs, which is consistent with findings in other species like *A. thaliana*, *B. rapa*, *Triticum aestivum*, and *Oryza sativa* and indicates their importance in floral development [11,12,16,19]. Specifically, 18 orthologs of *AtPERK5*, *AtPERK6*, *AtPERK7,* and *AtPERK12* were notably expressed in pollen, among which *AtPERK5* and *AtPERK12* were shown to be essential for pollen tube growth [11,27]. Furthermore, certain *B. rapa* genes (orthologs of *AtPERK6* and *AtPERK12*) were associated with male sterility, as they were downregulated in male-sterile mutants [19]. *BnPERK01A1* and *BnPERK01C1* (orthologs of *AtPERK1*) showed constitutively high expression, suggesting essential roles in fundamental cellular functions. Functional analysis of *BnPERK1* indicated its role in regulating cell wall integrity, signaling in stress responses, and facilitating growth and development in *Arabidopsis* [15].

Phylogenetic analysis indicated that the *BnPERK* genes originated through segmental duplication [28]. Structural examination confirmed the phylogenetic arrangement, with most *BnPERK* genes sharing conserved motifs and tyrosine kinase domains. Differences in gene structure and *cis*-acting regulatory elements in promoters suggested evolutionary divergence, which may contribute to functional diversity and evolutionary mechanisms [29]. Analysis of the *cis*-element indicated that *BnPERK* genes were responsive to various stresses and hormone signaling pathways, with MeJA- and ABA-responsive elements being particularly abundant. This suggests that *BnPERK* genes could mediate diverse gene expression patterns and provide flexibility in stress responses [30].

Further study on the expression patterns of *BnPERK* genes in response to hormone treatments provided insights into their potential roles in hormone signaling and plant development. This rapid and robust response to IAA, ET, GA, CK, and BL suggested that *BnPERK* genes might play a crucial role in the early stages of hormonal signal transduction in leaves, possibly by mediating the downstream effects of these hormones. In contrast, the expression profiles in roots exhibited totally different expression patterns, which highlights the complexity and specificity of hormonal regulation in different tissues. Enhanced expression levels after GA and ABA treatments in roots suggest involvements in root growth and stress adaptation [31,32]. The contrasting expression patterns after MeJA treatment in roots and leaves imply distinct roles in different tissues. Additionally, the observation that genes such as *BnPERK09A1*, *BnPERK09C1*, and *BnPERK04C1* were induced in both leaves and roots after IAA treatment implies a role in auxin signaling, which is central to various developmental processes, including cell elongation and differentiation [33]. These genes may act as intermediaries in auxin signal transduction pathways, influencing growth and development in a tissue-specific manner.

In summary, the comprehensive examination illuminates the structural diversity and functional implications of the *BnPERK* gene family. The differential expression patterns of *BnPERK* genes in response to various hormone treatments highlight their potential involvement in diverse physiological processes, including growth regulation, stress responses, and defense mechanisms.

### 3.3. Revealing the Role of BnPERK in Clubroot Disease

Recent studies have suggested that receptor-like kinases (RLKs) including PERKs are involved in plant–pathogen interactions and immune responses [2,3,4]. Analysis of *cis*-elements and expression profiles suggested that *BnPERKs* may act as novel sensors for various stresses, particularly plant pathogen infections [5,12]. Protein–protein interaction network analysis supported this result, revealing the multifunctionality of BnPERK proteins and emphasizing their broad participation in plant physiological processes specific to plant disease response [34]. The interactions involved cell wall organization, plant–pathogen interactions, plasma membrane dynamics, endomembrane activities, MAPK signaling pathways, and starch/sucrose metabolism. Given that pathogen-induced changes in cell walls affected disease resistance, it is possible that *BnPERKs* detect these alterations and initiate responses to maintain cell wall integrity [35]. The membrane localization of *BnPERKs* suggested they might perceive infection signals at the cell’s surface. Upon activation by cell wall components, *BnPERKs* likely trigger downstream signaling cascades, including MAPK pathways [36]. Otherwise, MAPK pathways also acted as downstream signaling components of hormones and played important roles in plant responses to different stresses [37]. Despite these insights, the exact role of *BnPERKs* in plant immunity remains largely unknown, and their interactions with RLKs or coreceptors remain largely unexplored.

Clubroot disease, caused by the obligate parasite *P. brassicae*, poses a significant threat to Brassicaceae plants [38]. The parasite is dependent on plant-derived sugars as a carbon source for colonization [39,40]. The enriched sugar metabolism reflects the altered homeostasis and increased demand for sugar in infected plants. However, the downstream signaling components and their regulation in response to *P. brassicae* infection are not yet clear. The *PERK* gene family, with its known functions in cell wall integrity and stress responses, may contribute to reinforcing the plant cell wall during pathogen infection or modulating defense-related signaling pathways [5]. Given the role of *PERKs* in disease response, the involvement of the *PERK* gene family in *B. napus* in clubroot disease was as expected. The transcriptional downregulation of *BnPERK09A1*, *BnPERK09C1*, *BnPERK14A1*, and *BnPERK06C2* upon *P. brassicae* infection suggests their putative roles in clubroot disease response. In accordance with the result in *B. napus*, the expression of *AtPERK9* was also downregulated in response to *P. brassicae*. The *perk9* mutant was used for clubroot disease investigation, indicating the roles of *BnPERK09A1* and *BnPERK09C1* in the disease resistance. Pathogenesis-related proteins including enzymes like β-1,3-glucanase (PR2) and thionins (PR5), which have antimicrobial activity and are involved in the basal immune response [41]. The *RBOHD* (Respiratory burst oxidase homolog D) gene is crucial in generating reactive oxygen species (ROS) as part of the plant immune system [42]. The increased expression of these genes suggested that the knockout of *AtPERK9* triggered the basal immune response, resulting in increased disease resistance in *perk9.* The induction of *PR2* and *RBOHD* in both Col-0 and *perk9* after inoculation suggested that basal immune response occurred in susceptible Col-0. Furthermore, the induction of *BnPERK09A1* and *BnPERK09C1* by IAA treatment was also particularly intriguing, as IAA is known to act as a signaling molecule potentially involved in the response to *P. brassicae* infection [43,44,45]. However, the exact roles of *BnPERK09A1* and *BnPERK09C1* need further function study. The predicted interaction proteins associated with these pathways implied their roles in mediating the interaction between *B. napus* and *P. brassicae*, providing targets for further functional characterization.

In summary, uncovering the role of *BnPERKs* in clubroot disease response provided valuable insights into plant–pathogen interactions and potential strategies for disease management.

## 4. Materials and Methods

### 4.1. Identification of BnPERK Genes in Brassica napus

The *BnPERK* gene family was selected in this study due to its critical role in plant growth, development, and stress responses. It has not been extensively studied in *B. napus*, making it a valuable target for genome-wide analysis. The genomic data and annotation for the *B. napus* cultivar ‘ZS11’ were sourced from *B. napus* multi-omics information resource (BnIR) (https://yanglab.hzau.edu.cn/BnIR, accessed on 1 March 2024) [46]. The *PERK* genes in *B. napus* were identified using BLASTp (https://blast.ncbi.nlm.nih.gov/) with an E-value threshold of 10^−5^, employing the protein sequence of 15 AtPERKs as queries [11]. The *BnPERK* gene family members were named based on their orthologous relationships and chromosomal location. By searching the conserved domain in InterPro (http://www.ebi.ac.uk/interpro, accessed on 5 March 2024) and the NCBI Conserved Domain Database (https://www.ncbi.nlm.nih.gov/Structure/cdd/wrpsb.cgi, accessed on 5 March 2024), the tyrosine kinase domain-containing protein sequences were verified for candidate *BnPERK* genes. Additionally, Expasy (https://web.expasy.org/protparam, accessed on 8 March 2024) was utilized to assess the isoelectric points (pIs) and the molecular weights (MWs) of BnPERK proteins. Subcellular locations of these proteins were predicted using Euk-mPLoc [47] (http://www.csbio.sjtu.edu.cn/bioinf/euk-multi-2, accessed on8 March 2024).

### 4.2. Chromosomal Location, Gene Duplication, and Phylogenetic Analysis

The positions of the *BnPERK* genes on the chromosomes were obtained from the annotation of the *B. napus* genome. McScanX (https://www.rcac.purdue.edu/software/mcscanx) was employed to detect the duplication patterns, including segmental and tandem duplication [48]. Visualization of chromosomal distribution and duplication events was represented using Circos (Vers. 0.69-9) software [49]. The Ka/Ks (nonsynonymous substitution rate to synonymous substitution rate) ratio was calculated for the paralogous gene pairs using ParaAT2.0 [50]. Collinearity analysis of *BnPERK* genes was conducted and displayed using TBtools (Vers. 2.056) [51]. A phylogenetic tree was generated using MEGA 11 [52] through neighbor-joining method with 1000 bootstrap replications and visualized using iTol (https://itol.embl.de, accessed on 11 March 2024).

### 4.3. Gene Structure, Conserved Motifs, and Cis-Elements Analysis

The exon–intron structure of *PERK* genes was examined using Gene Structure Display Server 2.0 (https://gsds.gao-lab.org/index.php/ accessed on 14 March 2024). Conserved motifs were predicted using Multiple Expectation Maximization for Motif Elicitation (https://meme-suite.org/meme/tools/meme, accessed on 14 March 2024). The sequences of 2 Kb upstream from the start codon of 50 *BnPERK* genes were extracted from the *B. napus* ‘ZS11’ genome and analyzed for *cis*-elements using PlantCARE [53] (http://bioinformatics.psb.ugent.be/webtools/plantcare/html, accessed on 14 March 2024) to predict *cis*-elements. The data were visualized using TBtools (Vers. 2.056) [51].

### 4.4. Expression Profiles of BnPERK Genes in Different Tissues and Under Hormone Treatments

To display different expression profiles, RNA-Seq data from various tissues and the hormone response of *B. napus* cultivar ‘ZS11’ were obtained from BnIR. A total of 14 tissues at different development stages included seed, leaf, bud, root, petal, sepal, pollen, silique, filament, cotyledon, lower stem peel, middle stem peel, upper stem peel, and vegetative rosette stages. The roots and leaves were treated with hormones (IAA, auxin; ACC, l-aminocyclopropane-l-carboxylic acid; GA, gibberellin acid; ABA, abscisic acid; TZ, trans-zeatin; JA, methyl jasmonate; BL, brassinolide) and sampled at 0.5, 1, 3, and 6 h after treatment. Heatmaps were generated using these expression values after logarithmic transformed RPKM values and displayed using TBtools (Vers. 2.056) [51].

### 4.5. Network-Based Prediction of Protein Interactions

Analysis of the protein–protein interaction network in *B. napus* was predicted using STRING (https://cn.string-db.org/, accessed on 15 March 2024), and the interactions were displayed using Cytoscape (Vers. 3.9.1) [54]. In order to investigate the biological process involving *B. napus* genes, the putative interacted genes based on Gene Ontology (GO) and Kyoto Encyclopedia of Genes and Genomes (KEGG) enrichment analyses were conducted by BnaOmics [55] (https://bnaomics.ocri-genomics.net/tools/enrich/, accessed on 15 March 2024).

### 4.6. Plasmodiophora Brassicae Inoculation, Clubroot Disease Investigation, and Histocytological Analysis

A collection of *Plasmodiophora brassicae* isolate from Zhijiang, Hubei, was used for inoculation in greenhouse. The seeds of *B. napus* cultivar ‘ZS11’, wild-type *A. thaliana* (Col-0), and *AtPERK9* mutant were germinated in a greenhouse maintained at 24 °C under a 16/8 h light/dark photoperiod. Seven-day-old rapeseed seedlings were inoculated with resting spores of *P. brassicae* at a concentration of 10^−6^ spores/mL [56]. Two-week-old *Arabidopsis* seedlings were inoculated at a concentration of 10^−5^ spores/mL. The clubroot disease was investigated and photographed at 28–35 days after inoculation. The disease index was assessed using the following 0–3 scoring system: 0 refers to healthy roots; 1 refers to small galls on lateral roots only; 2 refers to small and medium galls covering the main root and a few lateral roots; 3 indicates large galls on the main root. The disease index (DI) was calculated according to the following formula: DI = 100 × (1 × N_1_ + 2 × N_2_ + 3 × N_3_)/3 × N_t_. N_1_ to N_3_ refer to the number of plants in the indicated class, and N_t_ is the total number of plants tested.

The progression of cortex infection by *P. brassicae* was studied. Approximately 0.5 cm long root segments were excised from the upper of the primary root. Following standard protocols for fixation and dehydration, the samples were embedded in paraffin and sectioned at a thickness of 5–8 μM. Sections were analyzed and photographed using an optical microscope.

### 4.7. Transcriptome Analysis of BnPERK Genes in B. napus During Clubroot Disease

Root samples of *B. napus* infected with *P. brassicae* and uninfected controls were harvested at 14 days after treatment for RNA-Seq analysis. After sequencing and quality control, bioinformatics pipelines were using to clean raw sequencing data by removing adapters and low-quality reads. Then, the clean reads were aligned to the genome of ‘ZS11’. Gene expression levels were estimated using logarithmic transformed FPKM values. The differentially expressed genes (DEGs) between infected and control samples were determined with the DEGseq2 (adjusted *p* < 0.05 and |log_2_(fold-change)| ≥ 1) and displayed using TBtools (Vers. 2.056).

### 4.8. qRT-PCR Analysis of Pathogenesis-Related (PR) Genes in A. thaliana

Root samples from *Arabidopsis* Col-0 and *perk9* infected with *P. brassicae* and uninfected controls were harvested at 14 days after inoculation for qRT-PCR study. Total RNA was extracted from control and inoculated roots using the FastPure Universal Plant Total RNA Isolation Kit (Vazyme, Nanjing, China). First-strand cDNA was synthesized with HiScript IV RT SuperMix for qPCR (Vazyme). Gene-specific primers are listed in Supplementary Table S1. *Actin2* was used as reference gene. After running the qRT-PCR reactions, Ct (cycle threshold) values were obtained for each sample. The relative expression level was determined using the 2^−ΔΔCt^ method [57]. The data were presented as the mean ± standard error (SE) based on three technical replicates of three biological replicates. Significant difference was determined at a *p*-value < 0.05.

## 5. Conclusions

The study emphasized the evolutionary dynamics, structural diversity, and functional roles of the *BnPERK* gene family. It suggested that *BnPERK* genes are involved in various physiological processes, including growth regulation, stress responses, and the defense against pathogens. The transcriptional downregulation of *BnPERK09A1* and *BnPERK09C1* upon *P. brassicae* infection and the potential involvement of these genes in clubroot disease response further emphasize their significance in plant immunity. The identification of *BnPERK09A1* and *BnPERK09C1* as potential targets for disease management strategies and their putative role in signaling networks could contribute to the resistance improvement of Brassicaceae crops.

## Data Availability

Data are contained within the article and Supplementary Materials.

## Supplementary Materials

The following supporting information can be downloaded at: https://www.mdpi.com/article/10.3390/ijms26062685/s1.
