# Supplementary material for: Genome-Wide Analysis of the PERK Gene Family in Brassica napus L. and Their Potential Roles in Clubroot Disease
Source: Int J Mol Sci. 2025 Mar 17;26(6):2685. doi: 10.3390/ijms26062685 (PMC11942576; doi:10.3390/ijms26062685)
Supplement: Supplementary file 1 [file ijms-26-02685-s001.zip › Supp.pdf]

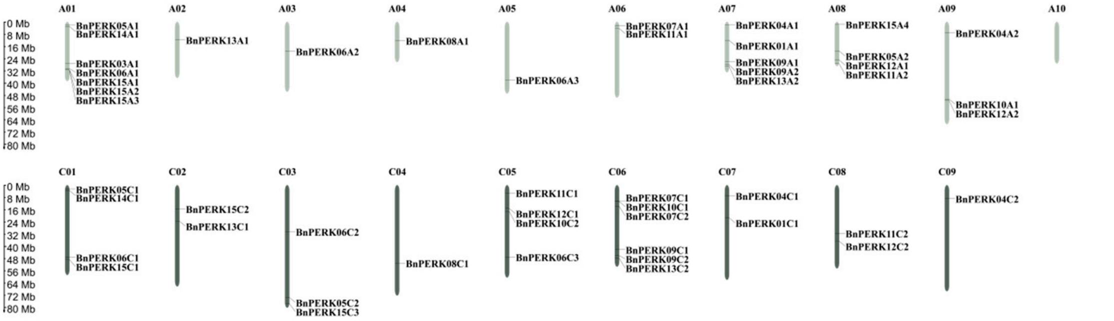

Supplementary Figure S1. Genomic distribution of identified *BnPERK* genes on the 19 chromosomes of rapeseed within A and C sub-genomes.

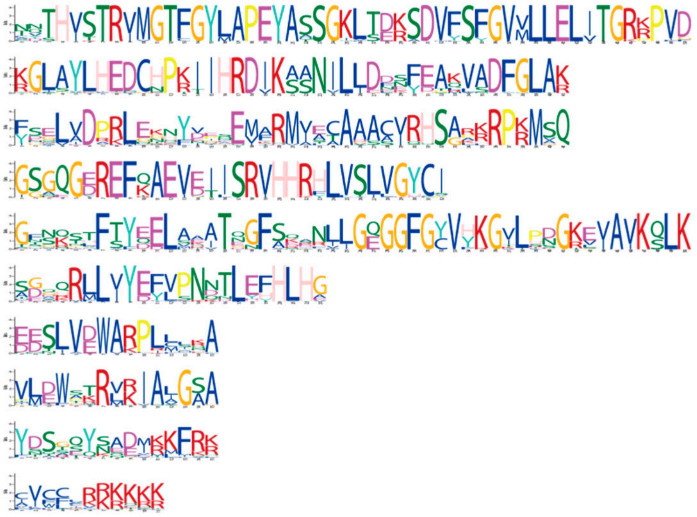

E-value	Sites	Width
1.6e-2148	48	50
5.4e-1759	49	41
3.9e-1363	47	41
7.4e-1341	46	32
1.1e-1616	45	50
2.1e-715	44	23
1.6e-439	47	15
1.1e-420	45	15
9.0e-320	44	15
8.1e-236	44	11

Supplementary Figure S2. Sequence logo conserved motif of the BnPERK proteins.

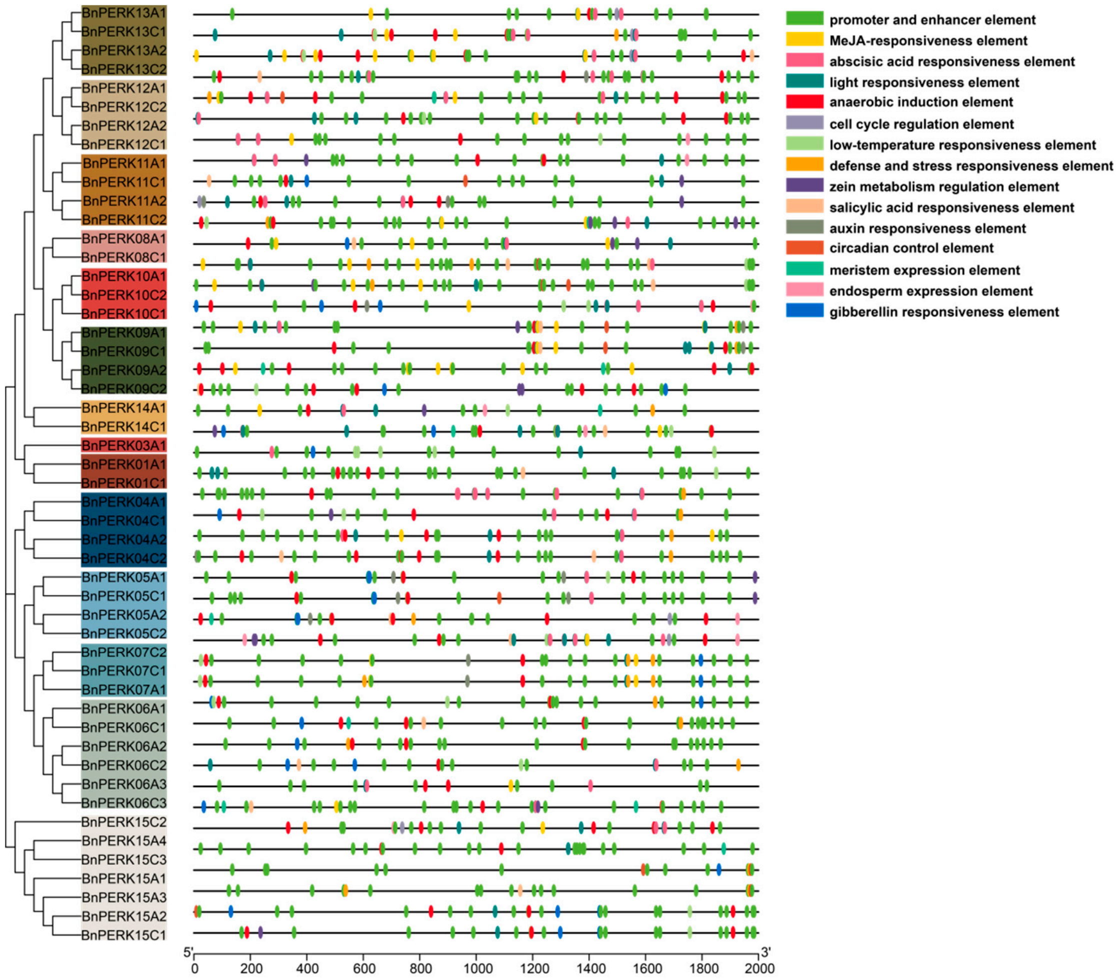

Supplementary Figure S3. *Cis*-elements analysis of the *BnPERK* promoters. The different colored boxes indicated various *cis*-elements present in the 2 Kb upstream regions of *BnPERK* genes.
